# Genetic characterization of Hawaiian isolates of *Plasmodium relictum *reveals mixed-genotype infections

**DOI:** 10.1186/1745-6150-3-25

**Published:** 2008-06-25

**Authors:** Susan I Jarvi, Margaret EM Farias, Carter T Atkinson

**Affiliations:** 1 Department of Biology, College of Arts & Sciences and Department of Pharmaceutical Sciences, College of Pharmacy, University of Hawaii at Hilo, 200 West Kawili Street, Hilo HI 96720, USA; 2 U.S. Geological Survey – Pacific Islands Ecosystems Research Center, P.O. Box 44, Hawai'i National Park, HI 96718, USA

## Abstract

**Background:**

The relatively recent introduction of a highly efficient mosquito vector and an avian pathogen (*Plasmodium relictum*) to an isolated island ecosystem with naïve, highly susceptible avian hosts provides a unique opportunity to investigate evolution of virulence in a natural system. Mixed infections can significantly contribute to the uncertainty in host-pathogen dynamics with direct impacts on virulence. Toward further understanding of how host-parasite and parasite-parasite relationships may impact virulence, this study characterizes within-host diversity of malaria parasite populations based on genetic analysis of the *trap *(thrombospondin-related anonymous protein) gene in isolates originating from Hawaii, Maui and Kauai Islands.

**Methods:**

A total of 397 clones were produced by nested PCR amplification and cloning of a 1664 bp fragment of the *trap *gene from two malarial isolates, K1 (Kauai) and KV115 (Hawaii) that have been used for experimental studies, and from additional isolates from wild birds on Kauai, Maui and Hawaii Islands. Diversity of clones was evaluated initially by RFLP-based screening, followed by complete sequencing of 33 selected clones.

**Results:**

RFLP analysis of *trap *revealed a minimum of 28 distinct RFLP haplotypes among the 397 clones from 18 birds. Multiple *trap *haplotypes were detected in every bird evaluated, with an average of 5.9 haplotypes per bird. Overall diversity did not differ between the experimental isolates, however, a greater number of unique haplotypes were detected in K1 than in KV115. We detected high levels of clonal diversity with clear delineation between isolates K1 and KV115 in a haplotype network. The patterns of within-host haplotype clustering are consistent with the possibility of a clonal genetic structure and rapid within-host mutation after infection.

**Conclusion:**

Avian malaria (*P. relictum*) and *Avipoxvirus *are the significant infectious diseases currently affecting the native Hawaiian avifauna. This study shows that clonal diversity of Hawaiian isolates of *P. relictum *is much higher than previously recognized. Mixed infections can significantly contribute to the uncertainty in host-pathogen dynamics with direct implications for host demographics, disease management strategies, and evolution of virulence. The results of this study indicate a widespread presence of multiple-genotype malaria infections with high clonal diversity in native birds of Hawaii, which when coupled with concurrent infection with *Avipoxvirus*, may significantly influence evolution of virulence.

**Reviewers:**

This article was reviewed by Joseph Schall (nominated by Laura Landweber), Daniel Jeffares (nominated by Anthony Poole) and Susan Perkins (nominated by Eugene Koonin).

## Background

Malarial infections consisting of mixed genotypes of the same species can be extremely common in human hosts, constituting over 80% of infections in high-transmission areas [1-3]. In rodent malarial infections, transmission rates of individual genotypes of *Plasmodium chaubaudi *are often higher from mixed infections than from single clone infections [4] and genetically distinct malaria clones compete within hosts [3]. Experiments with clonal lineages of *P. chaubaudi *have demonstrated that more virulent lines compete more successfully and that within-host selection can promote the evolution of virulence in malaria populations [5,6].

The *trap *gene encodes a cell-surface protein present in sporozoites and erythrocytic stage malaria parasites and was first described by Robson et al. [7]. The TRAP protein is critical for gliding motility (a unique behavior of apicomplexans that results in locomotion with no change in cell shape) and subsequent host cell invasion [7]. TRAP-deficient *Plasmodium *mutants are unable to invade either mosquito salivary glands or mammalian host hepatocytes [8]. The *trap *gene is a single-copy gene localized to chromosome 13 in *P. falciparum *based on hybridization of a *trap *probe to pulse-field gradient separations of intact chromosomal DNA molecules [9]. *Trap *paralogs have been identified in all motile *Plasmodium *life stages [10-12]. *Trap *orthologs have been found in many species within the phylum Apicomplexa including *Eimeria tenella *(EPT100) [13], *Cryptosporidium parvum *(TRAP-C1) [14], and *Toxoplasma gondii *(MIC2) [15]. Particular regions of the *trap *gene are critical for successful invasion [16] and survival [17]. Partial *trap *genes have been isolated from species of *Plasmodium *from avian hosts in Hawaii [18] and American Samoa [19]. This present study further characterizes diversity of the *trap *gene in isolates from avian hosts originating from three Hawaiian islands. Two isolates that were used in prior experimental infections were additionally characterized to begin to evaluate potential host effects on parasite diversity. We document the presence of multiple *trap *haplotypes of *P. relictum *within individual hosts, and define specific regions of interest within the *trap *gene for further population-level molecular studies.

## Results

Nested PCR was used to amplify a 1664 bp fragment from the *trap *gene from 18 malaria-infected birds originating from Hawaii, Kauai and Maui. All PCR products were subsequently cloned prior to analyses. These 18 birds included six Hawaii 'Amakihi (*Hemignathus virens*) which were experimentally infected with isolate K1 (originating from Kauai), five Hawaii 'Amakihi experimentally infected with isolate KV115 (originating from Hawaii) [20] and seven wild-infected birds of four species, 'Amakihi, I'iwi (*Vesteria coccinea)*, 'Apapane *(Himatione sanguinea) *and 'Elepaio *(Chasiempis sandwichensis)*, which originate from 3 islands (Hawaii, Kauai and Maui). Nested PCR was carried out using one of two enzyme/buffer systems. A total of 397 clones were obtained (Table 1). Of these, 357 *trap *clones were obtained using *Taq *polymerase (Promega) and 40 *trap *clones were obtained using FastStart High Fidelity PCR System (Roche). Also, 118 *trap *clones were obtained using standard PCR methods and 279 clones obtained using PCR+1 methods [21]. Additional details regarding the birds involved in this study, infection isolate and methodologies applied to produce clones are summarized in Table 1.

**Table 1:** Details regarding the samples include bird identification and species, isolate of *P. relictum *and island of origin of isolate; details regarding the methods include enzyme/buffer system and number of cycles for amplification, and method of amplification and cloning*

*Bird (DNA log)*	*Species*	*Isolate*	*Tissue Collection Date*	*Island*	*Enzyme*	*Number of cycles*	*Method*	*Total clones*	*Confirmed RFLP variants*	*Confirmed RFLP clones*	*Number of sequences*
1 (764)	*Hemignathus virens*	K1	Survivor Day 89 PI	Kauai	Promega	80	PCR+1	20	4	16	0
2 (1145)	*Hemignathus virens*	K1	Survivor Day 81 PI	Kauai	Promega	80	PCR+1	20	6	16	1
3 (1187)	*Hemignathus virens*	K1	Survivor Day 69 PI	Kauai	Promega	80	PCR+1	20	5	17	0
4 (584)	*Hemignathus virens*	K1	Fatality Day 16 PI	Kauai	Promega	80	standard	17	4	15	1
					Promega	80	PCR+1	20	5	18	1
5 (591)	*Hemignathus virens*	K1	Fatality Day 19 PI	Kauai	Promega	80	standard	9	4	9	0
					Promega	80	PCR+1	20	5	17	1
6 (593)	*Hemignathus virens*	K1	Fatality Day 19 PI	Kauai	Promega	80	standard	10	2	5	0
					Promega	80	PCR+1	20	5	16	0
					Promega	35	PCR+1	19	3	15	0
					Roche	35	PCR+1	20	2	20	10
7 (601)	*Hemignathus virens*	KV115	Fatality Day 16 PI	Hawaii	Promega	80	PCR+1	20	5	16	1
8 (613)	*Hemignathus virens*	KV115	Fatality Day 23 PI	Hawaii	Promega	80	standard	9	5	8	0
9 (736)	*Hemignathus virens*	KV115	Survivor Day 81 PI	Hawaii	Promega	80	PCR+1	20	8	17	4
10 (620)	*Hemignathus virens*	KV115	Fatality Day 24 PI	Hawaii	Promega	80	standard	17	7	17	0
					Promega	80	PCR+1	20	5	17	1
11 (623)	*Hemignathus virens*	KV115	Fatality Day 26 PI	Hawaii	Promega	80	standard	9	2	6	0
					Promega	80	PCR+1	20	4	13	0
					Promega	35	PCR+1	20	4	18	0
					Roche	35	PCR+1	20	2	18	10
12 (1757)	*Hemignathus virens*	wild	Blood 4/20/96	Kauai	Promega	80	standard	8	3	6	1
13 (1765)	*Vesteria coccinea*	wild	Blood 4/5/95	Hawaii	Promega	80	standard	9	5	9	1
14 (1818)	*Himatione sanguinea*	wild	Blood 3/28/95	Hawaii	Promega	80	standard	10	5	9	0
15 (1928)	*Hemignathus virens*	wild	Blood 11/16/93	Maui	Promega	80	standard	3	1	2	0
16 (2881)	*Chasiempis sandwichensis*	wild	Blood 2/3/95	Kauai	Promega	80	standard	9	4	8	1
17 (3186)	*Chasiempis sandwichensis*	wild	Blood 3/13/96	Kauai	Promega	80	standard	7	6	6	1
18 (6775)	*Hemignathus virens*	wild	Blood 4/24/01	Hawaii	Promega	80	standard	1	1	1	1

Totals								397		335	33

*Details regarding the clones include total number produced, number of confirmed (detected in > 1 bird) variants and clones, as well as the number of clones sequenced. Blood was collected from experimentally-infected birds that survived infection between days 69–89 post-infection (PI) when parasitemias had reached chronic levels. Pectoral muscle was collected from experimentally-infected birds that succumbed to infection between days 16–26 PI when acute phase parasitemias were high.

### RFLP Analysis

**Figure 1:**
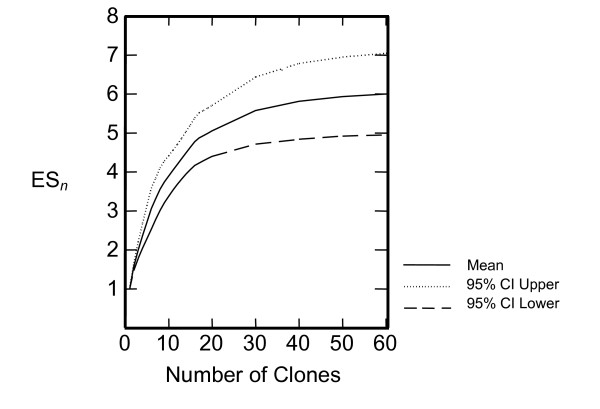
A rarefaction curve provides comparable estimates of species richness (ES_*n*_). The number of estimated haplotypes are plotted against the number of clones. The 95% confidence intervals are as indicated by dotted lines above (higher) and below (lower).

Amplified products from 397 *trap *gene clones were subjected to RFLP analysis using three enzymes (*Eco*RI, *Hpa*II, and *Taq*^α^I) and were analyzed by either agarose or acrylamide gel electrophoresis. A total of 88 distinct RFLP patterns (haplotypes) were detected, of which 28 distinct haplotypes were found among clones originating from multiple birds. Because these 28 distinct haplotypes were found independently in two or more birds, they are referred to as being "confirmed" *trap *haplotypes and represent the minimum number of haplotypes that are likely present in these infected birds (Table 2). One predominant RFLP haplotype (77) was found in all birds, and comprised 72% of all clones. Subsequent SSCP analysis of several clones with this predominant RFLP pattern revealed multiple additional banding patterns indicating further diversity among these RFLP-defined haplotype 77 clones (data not shown). Clone data from birds 15 and 18 were removed from further numerical summation due to minimal data collection with only 2 clones and 1 clone, respectively, obtained from these birds. In the 16 remaining birds, the minimum number of distinct *trap *haplotypes per bird ranged from 3 to 10, with an average of 5.9 haplotypes per bird. The average frequency of haplotype 77 was 0.63, but ranged from 0.17 in a wild 'Elepaio from Kauai to 0.80 in an 'Amakihi from Hawaii. We estimated diversity using the Simpson's index of diversity (D) [22] and the Shannon-Wiener index (H) [reviewed in [23]], and results are shown in Table 2. Diversity estimates by both indices produced parallel values and ranged from D = 1.49–6 and H = 0.69–1.79. These (generally) low parasite diversity values estimated in most individual hosts reflect the unequal abundances of haplotypes detected. One exception is 'Elepaio 17 from Kauai with D = 6, in which each haplotype was detected equally. Since the total number of distinct haplotypes detected is an indicator of within-host parasite variability and is likely dependent on the number of clones evaluated, we include the number of haplotypes/total clones ratio (hap/clones) as an additional reference indicator of variability. The hap/clone ratio varies among hosts ranging from 0.15 (in 'Amakihi 6 and 'Amakihi 11) to a value of 1.00 (in 'Elepaio 17). A rarefaction curve was generated in Primer v.5 [24] to provide comparable estimates of expected number of haplotypes (or expected species richness, ES_*n*_) based on number of clones evaluated (Figure 1). Mean values of ES_*n *_were plotted in Systat (vers 11.00.01), with 95% confidence intervals as indicated.

**Table 2:** Summary* of haplotype detection, diversity estimates and information regarding hosts infected with K1, KV115 or wild isolates.

	Isolate K1						Isolate KV115					Wild Isolates						
			
Bird ID	1	2	3	4	5	6	7	8	9	10	11	12	13	14	15	16	17	18
Species	H	H	H	H	H	H	H	H	H	H	H	H	I	A	H	E	E	H
Island	K	K	K	K	K	K	H	H	H	H	H	K	H	H	M	K	K	H
Survivorship	S	S	S	F	F	F	F	F	S	F	F	ND	ND	ND	ND	ND	ND	ND
1289 bp site	P	P	ND	P	P	P	A	ND	A	A	A	A	P	ND	ND	A	P	A
																		
Haplotype 1				1				1										
3		1		1														
4			2			1												
8					1			1		1				1		1		
10		1															1	
12	1	1		1	1	1			1							1	1	
20			2				1		1	1			2					
25						1					1		1					
27		1				3												
30								1		1			2				1	
33				1		1			1	1		1						
34					1		1									1		
36				1													1	
41					2			1		1								
44														1			1	
45			1						1		1							
47									1	2	1							
49									1		2							
51					1		1											
53	1									1								
56				1							1							
57				1			1		1	1								
58			1			1												
59					1						2			1				1
61	1									1				1				
62						1					3							
73		1										1						
77	13	11	11	26	19	47	12	4	10	24	44	4	4	5	2	5	1	0
Total Haplotypes	3	6	5	8	7	8	5	5	8	10	8	3	4	5	1	4	6	1
Total # clones	16	16	17	33	26	56	16	8	17	34	55	6	9	9	2	8	6	1
Freq. Hap 77	0.81	0.69	0.65	0.79	0.73	0.57	0.75	0.5	0.59	0.71	0.8	0.67	0.44	0.56	n/a	0.63	0.17	n/a
D (Simpson's)	1.49	2.03	2.21	1.59	1.83	1.41	1.73	3.2	2.7	1.97	1.55	2	3.24	2.79	n/a	2.29	6	n/a
H (S-W)	0.69	1.12	1.12	0.93	1.05	0.91	0.91	1.39	1.48	1.24	0.87	0.87	1.27	1.3	n/a	1.07	1.79	n/a
Hap/Clones ratio	0.19	0.38	0.29	0.24	0.27	0.15	0.31	0.63	0.47	0.29	0.15	0.50	0.44	0.56	n/a	0.50	1.00	n/a

*Birds are identified as in Table 1. Species are abbreviated as: H (Hawaiian 'Amakihi), I (I'iwi), A ('Apapane), and E (Elepaio). Island abbreviations are: K (Kauai), H (Hawai'i), M (Maui). Survivorship to malaria challenge is abbreviated as: F (fatality) and S (survivor). Designations at the 1289 bp site are either the amino acid P (proline) or A (alanine). ND (no data).

Average clonal diversity of isolates K1, KV115 and wild isolates, as well as those of clones originating from survivors and non-survivors are presented in Table 3. The diversity estimates of isolates K1 and KV115 are only marginally distinct from one another and this difference is not statistically significant by any index (described in Table 3, legend). Among the five wild isolates the overall average hap/clones ratio was 0.37, with those originating from Kauai having 10 haplotypes among 20 clones (hap/clones = 0.50), and those from Hawaii having eight haplotypes among 18 clones (hap/clones = 0.44). While diversity of clones originating from surviving birds are marginally higher than those of non-surviving birds based on all indices using similarily-generated clones (80 cycles, PCR+1 and Promega Taq), this difference was not statistically significant (Table 3, legend). Unique haplotypes were detected among isolates. Four of the 28 total haplotypes in Table 2 were unique to Kauai isolate K1 (3, 4, 27, and 58), and another three were detected in only K1 or wild Kauai isolates (10, 36 and 73). Only two of the 28 total haplotypes were unique to Hawaii KV115 (47 and 49) and not detected in any other isolate; only one of the haplotypes (44) was detected among wild isolates but not in either KV115 or K1. Five haplotypes (1, 41, 51, 56, and 62) were unique to non-survivors but not survivors or wild isolates, and an additional six haplotypes were present in non-survivors that were also present in wild isolates (8, 25, 30, 34, 36, and 59). Only two haplotypes (10 and 73) were found in survivors but not non-survivors and these were also detected among wild isolates.

**Table 3:** Summary of RFLP-defined haplotype diversity estimates* by isolate and survivorship.

	Isolates			Survivorship	
	KV115	K1	Wild	Survivors	Non-survivors
Number of birds	5	6	5	4	7
Haplotypes	20	24	14	16	18
Total # clones	130	164	38	66	97
Freq. Hap 77	0.72	0.77	0.55	0.68	0.92
D(Simpson's)	1.89	1.66	3.03	2.11	1.85
H(S-W)	1.37	1.2	1.97	1.45	1.32
Hap/Clones ratio	0.15	0.15	0.37	0.24	0.18

* Kruskal-Wallis Rank Test for K1 and KV115: for D, Mann-Whitney U test statistic = 9.000, P = 0.273, for H, Mann-Whitney U test statistic = 8.000, P = 0.201, for Hap/clones ratio, Mann-Whitney U test statistic = 8.000, P = 0.199. Kruskal-Wallis Rank Test for Surivors and Non-survivors: for D, Mann-Whitney U test statistic = 19.000, P = 0.345, for H, Mann-Whitney U test statistic = 16.000, P = 0.705, for Hap/clone ratio, Mann-Whitney U test statistic = 18.500, P = 0.393.

To examine the possible effects of PCR enzyme fidelity or specificity we performed side by side amplifications with 35 total cycles in two 'Amakihi (6 and 11) experimentally infected with isolates K1 and KV115, respectively, using standard *Taq *polymerase (Promega) and the FastStart High Fidelity PCR System (Roche) (Table 4). In both 'Amakihi 6 (isolate K1) and 'Amakihi 11 (isolate KV115), we observed diversity estimates greater (by all indices) among clones amplified with Promega *Taq *than in clones produced using the Roche system when other variables (35 cycles, PCR +1 methods) are held constant (Table 4, Trial 3 vs. Trial 4). In fact, the haplotype/clone ratio was reduced by 50% in both birds using the Roche enzyme system. We investigated the effect of cycle number on diversity by comparing 80 cycles vs. 35 cycles while holding other variables (*Taq *Promega polymerase, and PCR+1 methodology) constant (Trial 2 vs Trial 3). In both birds, we observed diversity values lower among clones generated with 35 cycles than among those generated by 80 cycles (by all indices). We also compared PCR + 1 amplification methods [21,25] with standard amplification and cloning methods in these two birds (Trial 1 vs. Trial 2). We observed greater diversity estimates of clones generated using PCR+1 methods as compared with those generated using standard methodology based on the Simpson's and Shannon-Wiener indices, but not the haplotype/clone ratio. Statistical analysis was not completed for any of these estimates in Table 4 due to low sample sizes (n = 2 birds) and are reported simply as empirical observations.

**Table 4:** Diversity estimate summation of side-by-side comparisons of enzyme/buffer system, cycling parameters and PCR methodology in two 'Amakihi infected with isolate K1 and KV115.

Bird ID Isolate	'Amakihi 6 K1 Kauai				'Amakihi 11 KV115 Hawaii			
Enzyme	Promega			Roche	Promega			Roche

Methods	Standard	PCR+1			Standard	PCR+1		
# cycles	80	80	35	35	80	80	35	35
	Trial 1	Trial 2	Trial 3	Trial 4	Trial 1	Trial 2	Trial 3	Trial 4
Haplotype 4			1					
12		1						
25		1					1	
27		2		1				
33	1							
45						1		
47							1	
49					1	1		
56							1	
58		1						
59								2
62			1			3		
77	4	11	13	19	5	8	15	16
Haplotypes	2	5	3	2	2	4	4	2
Total # Clones	5	16	15	20	6	13	18	18
Freq. Hap 77	0.8	0.69	0.87	0.95	0.83	0.62	0.83	0.89
D (Simpson's)	1.47	2	1.32	1.1	1.38	2.25	1.42	1.24
H (S-W)	0.5	1.04	0.49	0.2	0.45	1.03	0.63	0.35
Hap/Clone Ratio	0.40	0.31	0.20	0.10	0.33	0.31	0.22	0.11

### Sequence Analysis

Sequence information was obtained across the entire *trap *fragment (1624 bp, not including primer sequences) from 33 clones originating from 12 individuals (Table 1) and assigned Genbank nos. (EU760855-EU760887). Eighteen clones of the RFLP "dominant" haplotype 77 and fifteen clones representing 12 different haplotypes were sequenced. Two clones each were sequenced representing RFLP haplotype 12, and three clones of haplotype 59.

All sequences appeared highly similar to previously obtained *Plasmodium relictum trap *sequences [18,19]. Average nucleotide composition is 26% T, 16% C, 41% A, 17% G with an overall GC content of 33%. Overall *π *= 0.006 (0.001) (Tamura 3-parameter) (n = 33), with the ratio of nonsynonymous to synonymous nucleotide substitutions (*d*_*N*_/*d*_*S*_) = 1. Diversity of sequences representing clones of the dominant RFLP pattern variant 77 (n = 18) is *π *= 0.003 (0.001). Within-individual diversity is estimated at *π *= 0.003 (0.001) in both 'Amakihi 11 (n = 10, isolate KV115, Roche enzyme 35 cycles) and 6 (n = 10, isolate K1, Roche enzyme 35 cycles). In comparing the two experimental isolates, diversity of sequences originating from the K1 isolate (n = 13 among 4 birds) is *π *= 0.005(0.001), and of those from the KV115 isolate (n = 16 among 4 birds) is *π *= 0.006 (0.001); sequences from the four wild isolates (n = 4 among 4 birds) is *π *= 0.013 (0.002).

**Figure 2:**
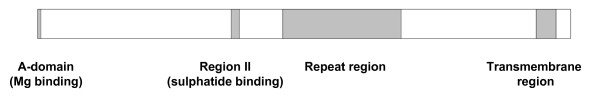
Schematic of a TRAP gene showing relevant domains.

**Figure 3:**
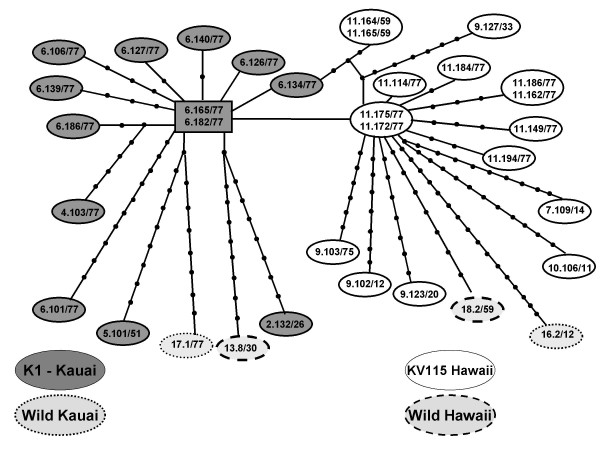
Haplotype network of the partial TRAP gene generated in TCS (vers 1.21). The maximum number of steps connecting parsimoniously two haplotypes is indicated by circular nodes. The haplotype with the highest outgroup probability is boxed, all others displayed as ovals. All haplotypes are represented by single sequences unless otherwise indicated by multiple sequence designations. The numerical sequence designations are as follows: bird number. clone number/RFLP haplotype designation.

A schematic of the *trap *gene of *P. relictum *is presented in Figure 2. These *trap *sequences are composed of functional domains similar to those previously described in other *Plasmodium *species [26,27] and in *P. relictum *[18,19]. The amino acid sequence corresponds to amino acids 56–533 of the *P. falciparum *clone T9/96 sequence [9]. An alignment file (Additional File 1) of the amino acid sequences (543 amino acids) is provided, and the nucleotide alignment file is available upon request. The core of Region II (which is also found in many other proteins) is WTPCSVTCG, which differs by one amino acid from that of other species of *Plasmodium *including *P. falciparum *[9] and *P. gallinaceum *[27] (WSPCSVTCG). The repeat region consists of 9 highly conserved repeats of 12 or 13 amino acids followed by a region of semi-conserved repeats between the repeat region and transmembrane region in Figures 2, and 3. The amino acid sequences (frequency) of repeats are as follows with the most highly conserved sites underlined and the cell-recognition signal RGD [9] conserved in three of the nine repeats in bold: repeat 1 PEDNR**RGD**VPDNV (1.00), repeat 2 PENKK**RGD**VPDYF (0.97), repeat 3 PEDNKPL-VPDNV (0.97), indel repeat PNNDPDNA (0.97), repeat 4 PENKK**RGD**VPDYF (0.94), repeat 5 PENNQPE-VPDNA (1.00), repeat 6 PEDNQPE-VPDNV (0.91), repeat 7 PEENQPE-VPYNV (0.94), repeat 8 PEENQPE-VPDNV (0.91), repeat 9 PEENQPE-VPDNV (0.97). Nucleotide polymorphism is localized to particular regions of the gene. The partial A-domain (Mg binding), the sufatide binding region (II), and the transmembrane regions are 100% conserved among all sequences. The transmembrane region is identical to that of *P. gallinaceum trap*, but differs from that of *P. falciparum *clone T9/96 by 3/19 amino acids. The 18 amino acids available from the cytoplasmic tail are highly conserved. Interestingly, the cytoplasmic tails are 100% identical in all sequences from non-survivors, but four of the five sequences from survivors have single amino acid changes (three of the four sequences from survivor 'Amakihi 9 (736) and one sequence from 'Amakihi 2(1145)). The sequence is terminated prior to inclusion of the clustered acidic residues shown to be important for aldolase binding [17].

Diversity across the entire sequence includes a total of 143 polymorphic nucleotide sites resulting in 87 amino acid changes. Deletions are detected at two sites in the 5' region (amino acids 124 and 130) and at one site in the repeat region (amino acid 170). The 13 polymorphic amino acid sites occurring in two or more sequences are of particular interest. Of these, five sites are dimorphic, involving only two amino acid substitutions. Site 539 bp (amino acid 179) is either K or E, site 570 bp (amino acid 189) is either H or R, sites 1178 bp (amino acid 392) and 1349 bp (amino acid 449) are either I or V. Of particular interest is site 1289 bp (amino acid 429), a G to C mutation, which results in a change from proline to alanine at the amino acid level. All sequences from a given individual, regardless of polymorphism at other sites, appear to be either proline or alanine at this site (specified in Table 2). All 10 sequences from 'Amakihi 11 and all 4 sequences from 'Amakihi 9 have alanine at this site, and all 10 sequences from 'Amakihi 6 have proline at this site.

The 33 sequences are presented in Figure 3 as a phylogenetic network [TCS,[28]]. The TCS software collapses sequences into haplotypes and calculates their frequencies. These frequencies are used to estimate haplotype outgroup probabilities, which correlate with haplotype age [28]. Ancestral haplotypes are identified by the internal positioning (shown boxed in Figure 3), and determined by degree of commonness and number of lineages derived from them [29]. Tip clades connected by only one connecting branch to the rest of the network are considered as derived. Based on these 33 input sequences, the ancestral haplotype is identified as originating from K1 (Kauai) isolate from 'Amakihi 6 and is represented by sequences 6.165 and 6.182. Three single step haplotypes or clades are derived from this ancestral haplotype, one of which (represented by sequences 11.175 and 11.172) forms a clade with most all other sequences from Hawaii (isolates KV115, and 4 wild Hawaii isolates). All other haplotypes involve multiple mutational differences (indicated by circular connections) with a maximum of 17. Within host diversity appears high with a total of nine haplotypes represented by 10 sequences from 'Amakihi 6 infected with isolate K1. Among birds infected with isolate KV115, seven haplotypes were represented by 10 sequences from 'Amakihi 11 and four haplotypes were represented by four sequences from 'Amakihi 9. The G/C polymorphism at nucleotide 1289 appears very influential in the network estimation as all haplotypes on the left side of Figure 3 have a C at this position and all those on the right side have a G in this position. Those on the left side consist of haplotypes from four 'Amakihi infected with K1 (Kauai), and one haplotype each from wild isolates from an Elepaio from Kauai and an I'iwi from Hawaii. Haplotypes on the right side originate from four 'Amakihi infected with isolate KV115 (Hawaii), and one haplotype each from wild isolates from an Elepaio from Kauai and an 'Amakihi from Hawaii.

## Discussion

The introduction of the mosquito vector, *Culex quinquefasciatus *to Hawaii in 1826 [30] provided a means of transmission for two introduced avian pathogens, avian malaria (*Plasmodium relictum*) and *Avipoxvirus*, that likely reached the islands in the late 19^th ^or early 20^th ^centuries [31-38]. At least two distinct genetic variants of *Avipoxvirus *are present in Hawaii, with one variant appearing more virulent than the other [38]. Recent studies have identified only a single lineage of *P. relictum *in Hawaii [39] previously identified as GRW4 [40] based on partial sequence data from the mitochondrial cytochrome b gene (n = 24 infected native birds) and nuclear DHFR-TS gene (n = 7 infected native birds).

We selected the gene encoding the cell-surface protein TRAP to target for analysis of within-host diversity of mixed genotype infections because of the critical role it plays in parasite locomotion and invasion of both the mosquito salivary glands and vertebrate host cells [7]. RFLP-based studies of *P. falciparum trap *clones revealed high levels of diversity with 96 haplotypes detected among 337 total clones [41] and sequencing of 73 clones from *P. vivax trap *revealed a total of 54 haplotypes [42]. All available information in other species of *Plasmodium *indicates that *trap *is a single-copy gene [9]. The organism is in the haploid state in the blood stage [reviewed in [43]], so the number of *trap *variants detected should be a strong indicator of the minimum number of genetically-distinct parasites present in an individual host. Our analysis includes comparison of experimentally-infected hosts which were exposed to the same parasite populations (same isolates) via single infective mosquito bites from mosquitoes originating from an inbred closed colony. Therefore, the differences in haplotypes detected among birds are likely reflecting host effects.

Multiple *trap *RFLP haplotypes were detected in all individuals ranging from two individuals having a minimum of three haplotypes to one individual having at least 10 distinct haplotypes. It is highly unlikely that this wide range of haplotype number among different isolates is due to the presence of multiple pseudogenes or that copy number differs so drastically in *P. relictum*. Sequence data further supports the presence of multiple haplotypes within individuals with nine distinct sequences representing 10 clones in 'Amakihi 6, seven distinct sequences representing 10 clones in 'Amakihi 11 and four distinct sequences representing four clones in 'Amakihi 9 (see Figure 3). Each isolate evaluated to date appears to be composed of a mixed-infection of genetically-distinct variants of *P. relictum*.

We report a minimum of 28 distinct haplotypes based on RFLP banding patterns. While overall we detected 88 RFLP-defined haplotypes, the other 60 variants were represented by only one individual clone and await further confirmation. Additionally, this initial survey was completed by RFLP analyses, which detects variation only at the restriction sites of the enzymes applied. This method inherently underestimates true variation. For example, while one RFLP haplotype (77) appeared predominant and was detected in all individuals, further evaluation by SSCP and sequencing revealed additional variation. The rarefaction curve also suggests we have underestimated diversity, indicating a more reasonable estimate might require substantial sampling (up to 30–40 clones per individual). The 28 haplotypes reported here thus represent a minimum number existing in Hawaiian parasite populations at the time of sample collection.

We evaluated several technical aspects of the study in a side-by-side comparison in two birds. Because *trap *is a single-copy gene and in relatively low concentrations in a typical blood sample from an infected bird, nested PCR was necessary to produce enough product for visualization on an agarose gel. While nested PCR increases specificity and sensitivity of detection, a greater number of cycles, and therefore a higher risk of enzyme error, is inherently applied in the process. A main difference between the two enzyme/buffer systems used is that the FastStart High Fidelity PCR System (Roche) includes a proofreading enzyme whereas Taq polymerase (Promega) does not. The enzymes also require different buffers, which may influence primer specificity. We initially used the Promega enzyme to ensure the presence of an A overhang for downstream cloning. We found that both a decrease in the number of cycles from 80 to 35 and the use of the Roche system vs. the Promega system reduced the diversity of clones generated, although the cause of this is not entirely clear. We lessened risk of including artifactual clones generated by nucleotide mis-incorporation by the enzyme by excluding RFLP variants that were not confirmed by independent replication in a second bird. While it is highly unlikely that random mis-incorporation would occur at least twice at same restriction site, it is not impossible. Other possibilities may be that the higher variability observed at 80 cycles (with enzyme error set aside) is the result of amplification of rare *trap *haplotypes that could be missed using fewer cycles. Or, the differences in variability observed between different enzyme/buffer systems might be due to a change in primer specificity due to the differing buffer recipes. Similarly, but in a different study, we observed only minimal overlap in the sets of major histocompatibility complex (*Mhc*) class II alleles amplified from birds when using the Promega enzyme/buffer system versus when using the Roche system (Jarvi et al., ms. in prep). A change in specificity might be expected and is observed using PCR+1 techniques vs. standard methods due to incorporation of the M*luI *site used for selection of homoduplexed PCR products. The addition of these four nucleotides may alter specificity, resulting in amplification of the greater numbers of distinct *trap *haplotypes observed in both birds.

The *P. relictum trap *gene possesses hallmark characteristics previously defined in other *Plasmodium spp*. [9,27]. The core amino acid motif of region II (sulfatide-binding region) is 100% conserved in all *P. relictum *sequences, but all *P. relictum *TRAP sequences possess a single amino acid substitution (Thr instead of Ser) at the second site of this region as compared with TRAP from other species of *Plasmodium*. The transmembrane region is 100% conserved among our and other *Plasmodium *TRAP sequences. Also similar to other TRAP sequences, we detected multiple highly conserved (> 90%) repeat units of which three possess the conserved cell-recognition signal (RGD). While the degree of conservation of these main characteristics among *trap *genes from varying *Plasmodium spp*. appears high, the biological significances of the changes detected in *P. relictum *TRAP remain unknown.

The high diversity of *trap *suggested by RFLP was confirmed by sequencing with detection of 143 polymorphic sites resulting in 87 amino acid changes. The extent of diversity missed by RFLP analyses is clearly demonstrated by phylogenetic network analyses of sequences (Figure 3) with multiple haplotypes found which were initially all assigned by RFLP as haplotype 77. Overall diversity of sequences is low (*π *= 0.006/0.001), and is even lower within haplotype 77 sequences (*π *= 0.003/0.001), but 18 of the total of 19 RFLP haplotype 77 clones sequenced proved to be distinct at the nucleotide level.

A primary focus of this study is comparison of the parasite populations from two isolates, one from Kauai (K1) and one from Hawaii (KV115). These isolates were originally collected from the wild; isolate KV115 originated from an 'Apapane from Kilauea Volcano area on the Big Island of Hawaii in 1992, and isolate K1 from Elepaio from the Alakai Wilderness Preserve on Kauai in 1995. These original samples are not currently available for evaluation. Also, unlike the situation in primate malarias, *P. relictum *has not yet been successfully cultured in the lab, thus all studies are based on wild isolates. While overall clonal diversity levels did not appear different between isolates K1 and KV115, we detected a higher number of unique RFLP clones in isolate K1 than in isolate KV115 (Table 2), and the sequenced haplotypes from K1 and KV115 appear clearly distinct from one another in the phylogenetic network (Figure 3). The haplotype network analysis provides a means of inference of an ancestral haplotype (which is boxed in Figure 3, a Kauai K1 haplotype) as compared with those which are derived. If K1 is typical of the parasite population on Kauai (which has yet to be determined), it could be that malaria parasites colonized Kauai prior to Hawaii and have had more time to evolve (with the ancestral type from Kauai and the higher number of unique haplotypes so far found there). The higher number of unique RFLP haplotypes found in K1 may also reflect differences in selective pressures on these isolates, or may simply be indicative of the founding population of parasites on each island. But, while this initial analysis provides important baseline information regarding diversity within an individual host, it is not currently sufficient to project to geographic populations. A population level analysis is currently underway (ME Farias, Master's thesis, University of Hawaii at Hilo).

Clonal diversity was high as well within individual hosts based on RFLP and sequence data from multiple clones from three individual 'Amakihi (6, 11 and 9). A recent study in lizards naturally infected with *P. mexicanum *supports data found with *P. falicparum *in that malaria parasites maintain high genetic diversity in host populations even during times when transmission intensity is low [44]. Our data in birds supports the supposition of high clonal diversity as a general phenomena of malarial parasites. Whether this high diversity was present at the time of introduction to the islands, evolved over time, or is due to rapid mutation within individual infections, PCR error, or combinations of these factors has yet to be determined. We minimized the inclusion of clones resulting from PCR error by using only those detected independently in two different individuals. While it is highly unlikely that random error would occur at the same sites, *trap *could be a gene very prone to copy errors. However, a high PCR error rate is not consistent with our sequence data (Additional File 1) as the regions we expect to be conserved (e.g. the transmembrane region and others) are in fact highly conserved. The relationships revealed in the haplotype network indicate a more likely scenario. There is clear delineation between haplotypes of K1 versus KV115, regardless of host. Within hosts (i.e., Amakihis 6 and 11) haplotypes tend to cluster relatively closely together and have one central haplotype from which others are derived. Also, data suggests a single lineage of *P. relictum *exists in Hawaii based on mtDNA genes [39]. We have no definitive data on clonal diversity levels at the time of introduction to the Hawaiian islands, so we can not address these comparisons at this time, but our findings with the *trap *gene appear consistent with the possibility that rapid mutation is occurring within each host, most likely after infection with a single or relatively few initial clones (clonal genetic structure). We know within-host selection may play a role in the generation of new variants. While we did not find strong evidence for selection in our limited dataset (*trap *sequences display a higher number of nonsynonymous substitutions then synonymous, but when normalized for synonymous substistutions, the *d*_*N*_/*d*_*S *_value equals 1), previous studies in human malarias from more geographically-diverse samplings have reported evidence of positive selection on *trap *in *P. falciparum *[9] and *P. vivax *[42]. The same finding in our ongoing population-level studies would provide one mechanism for generation of within-host diversity.

We detected 87 amino acid changes with 13 sites occurring in two or more sequences. These polymorphic sites form the basis of our SNP-based analysis of *trap *diversity in wild populations. Of the five dimorphic amino acid sites detected, site 430 (*trap *nucleotide position 1289) appears to be particularly interesting. All clones from a single host bird are either G or C at 1289, none of the birds evaluated had a mixture of alleles at this position. This polymorphism appears to be isolate-specific, and not dependent on geographic origin (both polymorphisms are present in wild birds from both Hawaii and Kauai), and, at first glance, does not appear related to survivorship as both are present in survivors and non-survivors, but the resulting amino acid change, from alanine to proline, could have a significant impact on protein folding and function.

We know from previous studies that some birds survive while others do not when infected with either K1 or KV115 [20]. If *trap *is a determinant of virulence we would expect that some haplotypes would be present in survivors and not in non-survivors and vice-versa, and this is what we find. We also observed slightly (but not significantly) higher levels of clonal diversity among parasite populations from survivors, with some of that diversity localized to the cytoplasmic tail. Studies of mixed-genotype malarial infections indicate that transmission rates of individual genotypes are often higher from mixed rather than from single-clone infections [4]. Higher clonal diversity could be a significant driving force for both increasing transmission rates and moderating parasite virulence.

## Methods

### Isolates of P. relictum

The two laboratory isolates of *P. relictum *that were used in this study originated from wild hosts on Kauai or Hawaii Islands. The KV115 isolate was obtained in 1992 from a wild 'Apapane (*Himatione sanguinea*) captured at Kilauea Crater in Hawaii Volcanoes National Park [33]. The K1 isolate was obtained from pooled blood samples from three Elepaio (*Chasiempis sandwichensis*) captured in the Alakai Wilderness Preserve in August 1994 [20]. Blood from only one of the three Elepaio was later found to have a detectable parasitemia by microscopy. Fresh, heparinized blood from wild hosts was inoculated intravenously into individual canaries (passage 1) to amplify the number of parasites prior to aliquoting and cryopreservation of the isolates.

The KV115 isolate was passed once in a canary prior to freezing. Several weeks prior to infection of 'Amakihi in this study, an aliquot was thawed, deglycerolized, and inoculated intravenously into an uninfected canary (passage 2). When parasitemia was detectable by blood smear, approximately 0.1 cc of heparinized whole blood was passed intravenously into a one week-day-old Pekin Duckling (passage 3), passed two more times into Pekin Ducklings to amplify parasitemia and then inoculated into three Pekin Ducklings (passage 6) that were used to infect mosquitoes (*Culex quinquefasciatus*) as described by Atkinson et al. [33].

After initial recovery in a canary (passage 1), The K1 isolate was passaged into a second canary (passage 2), and 4 Pekin Ducklings (passages 3–6) prior to aliquoting and cryopreservation. Several weeks prior to infection of 'Amakihi, an aliquot was thawed, deglycerolized and inoculated intravenously into a Pekin Duckling (passage 7) and subsequently passaged two more times in Pekin Ducklings (passages 8 and 9) to increase parasitemia prior to infection of mosquitoes.

### Sample collection

#### Experimental infections

Adult Hawaii 'Amakihi were captured with mist nets in January 1995 in high elevation xeric habitat in the Mauna Kea Forest Reserve, transported to a screened mosquito-proof aviary at Hawaii Volcanoes National Park and acclimated to captivity as described elsewhere [34,20]. Birds were exposed to single infective mosquito bites from individual *Culex quinquefaciatus *that were reared in a closed colony and infected with either the K1 or KV115 isolates of *P. relictum *as described by Atkinson et al. [20]. Five birds were infected with the KV115 isolate from the island of Hawaii and 6 Hawaii 'Amakihi were similarly exposed to the K1 isolate of *P. relictum *from the island of Kauai. All birds were bled via the brachial vein for preparation of blood smears every 4 days or via the jugular vein for collection of DNA samples. Birds that died during the course of infection from acute malaria were necropsied to verify cause of death. Blood samples from live birds and pectoral muscle tissue taken during necropsy were stored frozen in lysis buffer until DNA was extracted [18].

#### Natural infections

Wild birds were captured with mist nets in the Alakai Wilderness Reserve on Kauai, in the Hanawi Natural Area Reserve on Maui, or Kona Unit of Hakalau Forest National Wildlife Refuge and Nanawale Forest Reserve on the island of Hawaii. Approximately 100 μl of blood was collected via jugular venipuncture into a heparinized 1.0 cc insulin syringe, transferred to microhematocrit tubes and centrifuged to collect plasma. Packed red blood cells were transferred to lysis buffer and stored frozen until DNA was extracted. Summation of all samples, origin, and dates of collection are described in Table 1.

### DNA isolation

Genomic DNA was extracted from tissue samples using standard techniques. Briefly, samples were digested in 1 ml of 50 mM Tris-HCl, 100 mM sodium EDTA, 2% SDS with 0.5 mg/ml proteinase K overnight at 55°C. DNA extractions were completed with one phenol, two phenol/dimethyl chloride 1:1, and one dimethyl chloride/isoamyl alcohol (24:1) extraction steps, and dialyzed against 10 mM Tris-HCl, pH 8.0, 1 mM EDTA. DNA concentration was quantified by spectrophotometry or agarose gel electrophoresis.

### Nested PCR amplification of the trap gene

#### Standard PCR

Between 250 and 1000 ng of genomic DNA was used as template in a 25 μl reaction containing 1.6 μM each primers P1 (GAT GAA ATA A^A^_C_^A^_T _TA^C^_T_^A^_C_^A^_G_T GAA ^C^_G_AA ^A^_G_T^A^_T _TGT) and P2 (CCA ^A^_C_TC ^A^_G_TT ^A^_T_TC TTC ^A^_T_GG TAA) (Jarvi et al. 2002), 1× reaction buffer, 4 mM MgCl_2_, 0.8 mM total dNTPs, and 1.25 units Taq DNA polymerase (Promega). Samples were subjected to 40 cycles of 94°C for 30 seconds, 45°C for 1 minute, and 72°C for 2 minutes. 5 μl of product from the first reaction was then used as template in a 50 μl reaction containing 0.8 μM each primers P5 (GACCTTTATATACTAATGGATGG) and P6 (CCTTCACCAAGTACATCATT) [18], 1× reaction buffer, 2 mM MgCl_2_, 0.6 mM total dNTPs, and 1.67 units Taq DNA polymerase. Cycling parameters were 40 cycles of 94°C for 30 seconds, 50°C for 1 minute, and 72°C for 2 minutes. PCR products were analyzed by electrophoresis on 1.5% agarose gels (SeaKem, FMC Bioproducts) and reactions producing a band at approximately 1650 bp were considered to be successful. Products were purified on a low melting point agarose gel (see below) and stored at -20°C until cloning.

#### PCR + 1 amplification

In order to eliminate the possibility of heteroduplex formation, PCR + 1 methods as described by Boriello and Krauter [21] were used. The first reaction took place as described above, except that only 0.8 μM each primers P1 and P2 were used. 3 μl of this product was used as template in an asymmetric nested PCR reaction consisting of 0.4 μM primer P6 and 0.04 μM primer P5. Remaining parameters were as described above for the second reaction. The approximately 1650 bp product was then run on a 1.5% low melting point agarose gel, excised, and gel purified using the QIAQuick Gel Extraction Kit (Qiagen, Inc.) according to manufacturer's instructions. Between 12 and 20 μl of purified product was used as template in a 50 μl reaction volume containing 0.8 μM primer P5.MLU (5' ACGCGTGACCTTTATATACTAATGGATGG 3') only, with remaining reaction components as above. Samples were subjected to a single cycle of 94°C for 1 minute, 50°C for 2 minutes, and 72°C for 10 minutes. Samples then were stored at 4°C or -20°C until cloning.

#### FastStart High Fidelity PCR + 1 amplification

Side by side amplifications were performed using Taq polymerase (Promega) and the FastStart High Fidelity PCR System (Roche). Conditions for the Promega reactions were as described for the PCR + 1, except that the final volume for the second reaction was reduced to 25 μl. The number of cycles was also reduced to 15 in the first reaction and 20 in the nested reaction. The Roche first reaction contained 1.0 μM each primers P1 and P2 [18], 1× reaction buffer, 2 mM MgCl_2_, 0.9 mM total dNTPs, and 1.25 units Fast Start High Fidelity Enzyme Blend (Roche). The Roche nested reaction contained 0.4 μM primer P6, 0.04 μM primer P5, 1× reaction buffer, 2 mM MgCl_2_, 0.6 mM total dNTPs, 4% DMSO and 1.25 units Fast Start High Fidelity Enzyme Blend (Roche). All other conditions were identical to those described for Promega reactions in this section. The single PCR + 1 cycle for both Promega and Roche reactions utilized Taq DNA polymerase (Promega) to ensure incorporation of the A-overhangs necessary for TA cloning.

### Cloning of PCR Products

Gel-purified PCR products or PCR + 1 products were ligated into the pCR 2.1-TOPO vector and transformed into TOP10F' *E. coli *using the manufacturer's suggested protocol (TOPO TA Cloning Kit, Invitrogen). Colonies were screened by PCR amplification using primers P5 and P6 for regular PCR products or plasmid primers M13F20 and M13R (Invitrogen) for PCR + 1 products. PCR + 1 colonies were further screened by digestion of the M13 PCR product with the restriction enzyme *MluI*. Plasmid DNA was obtained using the QIAprep Mini Kit (Qiagen) according to the manufacturer's suggested protocol.

### Amplification of cloned fragments for RFLP analysis

For non-PCR+1 products, the cloned *trap *gene fragment was amplified directly from the colonies. Cells picked directly from a single colony were added to a PCR reaction containing 1.6 μM each primers P5 and P6, 1× reaction buffer, 3 mM MgCl_2_, 0.8 mM total dNTPs, and 1.67 units *Taq *DNA polymerase (Promega). The reactions were heated at 94°C for 1 min, then subjected to 40 cycles of 94°C for 30 seconds, 50°C for 1 minute, and 72°C for 2 minutes. For PCR+1 products, the cloned *trap *gene fragment was amplified from the M13 PCR product used for colony screening or from plasmid DNA. 1 μl of PCR product diluted 1:100 or 1 μl of plasmid DNA diluted 1:200 was used as template in a 50 μl reaction containing 0.4 μM each primers P5 and P6, 1× reaction buffer, 2 mM MgCl_2_, 0.6 mM total dNTPs, and 1.25 units *Taq *DNA polymerase (Promega). Cycling parameters consisted of 5 cycles of 94°C for 30 seconds, 65°C for 1 minute, and 72°C for 1 minute, followed by 5 cycles with an annealing temperature of 63°C and 20 cycles with an annealing temperature of 60°C. Denaturing and extension times and temperatures did not change.

### RFLP analysis

P5 and P6 PCR products (5 μl) amplified from clones were digested in a total volume of 10 μl with *Eco*RI (10 units), *Hpa*II (10 units), or *Taq*^α^I (20 units) in corresponding 1× reaction buffer (New England BioLabs). The *Taq*^α^I digestion also contained BSA at a concentration of 100 μg/ml. Digestions were incubated at 37°C (*Eco*RI, and *Hpa*II) or 65°C (*Taq*^α^I) for 90 minutes, then stored at 4°C prior to RFLP analysis. All digested *trap *fragments were run on 2% agarose gels (3 NuSieve:1 SeaKem, FMC Bioproducts) in 1× TBE buffer at 100 V for 1 hour and 15 minutes with 100 bp DNA ladder (New England Biolabs) every 13 lanes. Further resolution of some clones was achieved by electrophoresis on 10% non-denaturing acrylamide gels in 1× TBE (PAGEr Gold, Cambrex) at 150 V for 1 hour and 20 minutes. TIFF images of ethidium bromide-stained gels were scanned and imported into BioNumerics v. 3.5 (Applied Maths). The gels were normalized using the ladder and common bands. Automatic band calling and band matching were hand corrected.

### Sequencing

In most cases, clones were chosen for sequencing based on variable RFLP banding patterns. In the case of the Roche PCR products, all clones showing RFLP variation were sequenced, and an additional subset of clones was randomly selected so that a total of 10 clones from each individual were sequenced. Sequencing reactions contained 50–100 ng plasmid DNA. Sequencing primers were: M13F20 and M13RV, modified plasmid primers M13F20X and M13RVX [45], *trap *primers P3, P5, P6 [18], TPSEQ4.5 (5' GACTGTTCTCCTTAT 3'), TPSEQ5 (5' GCCTTAAGTAGAGTATT 3'), CEQTP3.1 (5' CCTTATAATCATTTCTCCTTGGTCGT 3'), CEQTP5.1 (5' AGGAAGAGAAGACGCGGTTCAA 3') and CEQTP5.2 (5' GATGTTCTGATCACATGAATGCTCTT 3'). Sequencing was completed on an ABI 373-cycle sequencer (BMBITF/PBRC, University of Hawaii at Manoa, Honolulu, HI) or a CEQ 8000 capillary electrophoresis system (Hilo Genetics Core Facility, University of Hawaii at Hilo, Hilo, HI). Sequences were proofread, assembled and aligned using Sequencher 3.0 (GeneCodes Corp). Alignments were imported into MEGA version 3.1 [46] for further analysis.

### Diversity

For RFLP data, diversity was estimated using the Simpson's Index [22] with the formula D = 1/∑*p*_*i*_^2 ^and the Shannon-Weiner Index H = -∑*p*_*i *_log_e _*p*_*i *_reviewed in [23]. A rarefaction curve was generated in Primer v.5 [24] with mean values of expected species richness ES_*n *_plotted in Systat (vers 11.00.01). Statistical comparisons of diversity estimates were also completed in Systat (vers 11.00.01). Sequence data was used to produce a phylogenetic network using statistical parsimony to connect the haplotypes based on 95% confidence interval (TCS 1.21) [28]. Distance analyses (Tamura 3-parameter with pairwise deletion) of sequence data were conducted using MEGA version 3.1 [46].

## Competing interests

The authors declare that they have no competing interests.

## Authors' contributions

SIJ contributed to the design, analysis, and interpretation of the data and served as primary author for manuscript preparation.

MEMF contributed to the technical acquisition of data and provided valuable insights toward the drafting of the manuscript.

CTA provided the biological materials for analysis, statistical contributions and contributed greatly to the design and interpretation of data and drafting of the manuscript.

All authors have read and approved the final manuscript.

## Reviewers' comments

Review 1 (Joseph J Schall, University of Vermont, Department of Biology, Burlington VT, USA; nominated by Laura Landweber, Dept. of Ecology and Evolutionary Biology, Princeton University, Princeton, NJ, USA)

The arrival of the avian malaria parasite *Plasmodium relictum *on the Hawaiian islands in the early 20th century (about century after a competent vector was introduced by accidental human action) led to an ecological catastrophe of almost biblical proportions. Many endemic bird species were reduced in density, altered in their distribution, or driven to extinction. The phenomenon of the bird community was destroyed. Apart from the heartbreaking esthetic loss, these events argue that pathogens may have always played a role in shaping biodiversity. Even rare such events would have long-term importance. And, the Hawaiian bird malaria story warns us the emerging infectious disease can have the broadest consequences.

The Hawaiian bird malaria team has produced a string of reports that rank among the most important in the history of parasitology. Here, the team members examine the genetic diversity of the TRAP gene. TRAP is an important surface protein involved in the weird gliding movement of apicomplexan cells.

The authors report three general findings:

1. The structure of the gene and protein is characterized in some detail.

I have only limited experience with such analysis, and so can offer no informed comments.

2. There is some relationship between virulence and allele diversity of TRAP per host. The manuscript brings up "host survivorship" [Abstract line 38] or "surviving birds" [first mention on line 136]. What this means is not provided until line 431/432, so this survivor issue drops out of nowhere. Also, there were only 18 birds under study, so with this small sample size, nothing can be inferred about the allelic diversity of TRAP and survival of experimental birds. Therefore, I will not pursue this issue here, and feel that the topic should not be included in an otherwise fine presentation.

**Authors' response**: This is quite right. This manuscript is intended as a genetic analysis of several individual hosts to begin to characterize diversity and develop additional methods for future population-level analysis based on *trap*. It not at all intended as an analysis of survivors and non-survivors of avian malaria. However, many of the individuals under study were involved in experimental infection studies. The author's feel compelled to include this data here, but we have reworked it so as to more accurately reflect its somewhat minimal significance.

3. There is a substantial diversity of TRAP alleles even within single infections. At least 88 alleles are identified, and this appears to underestimate the true diversity. Of these, 28 appear in more than one bird examined, and are thus given the closest attention. This allelic diversity could be a result of (i) a large number of genotypes of the parasite arriving over the past decades; (ii) mutation of the TRAP gene leading to diversity after the parasite became established; (iii) a combination of (i) and (ii); (iv) rapid mutation of TRAP within individual infections leading to new variants even if an infection began with a single allele; (v) PCR artifacts leading to an apparently high diversity. My reading of the results comes to a different outlook on the story than the authors', but this different conclusion would still would be a very interesting and important.

Malaria researchers for many years have suspected that the surface proteins of *Plasmodium *may be under selection to mutate rapidly. Diversity may be favored in response to the attack of the immune system. Previous studies on the malaria parasites of humans have found a very large number of alleles for surface protein genes cycling in a single population (84 alleles detected in one study, similar to the results for TRAP in *P. relictum)*. A very rapid mutation rate may even lead to new alleles appearing during the course of a single infection.

The "Conclusions" begin with the statement: "This study shows that the genetic diversity of Hawaiian isolates of *P. relictum *is much higher than previously recognized. These results indicate the widespread presence of multiple-genotype infections in the native birds of Hawaii." But, another conclusion could be reached. If there is very rapid mutation of TRAP (at least the variable portions of the gene), we might imagine a clonal genetic structure, with no variation in the genome except for the genes coding for surface proteins. That is, case (iv) and even case (v) above may be the situation.

**Authors' response**: These are valid points. We have now included a phylogenetic network analysis (at the suggestion of another reviewer) from which we were able to provide a clearer presentation of the relationships of (sequenced) haplotypes. These data appear consistent with (iv) above, rapid mutation, and have now been included.

The number of infections studied was 2 from lab birds (from two infections collected in nature, I assume (isolates KV115 and K1), and 7 more wild-caught birds. So, we have 9 infections under study. From those 9 infections, there are 80+ alleles. Also reported is that more alleles are detected if the PCR is run for 80 cycles vs. 35 cycles. This last result suggests that mutations are taking place during the PCR. After all, PCR replicates what happens in cells, so this seems to indicate that the gene is very prone to copy errors.

**Authors' response**: While one can never totally eliminate the possibility of random PCR error, we took several steps to minimize inclusion of clones with higher potential for error. We only included clones produced independently in two or more birds under the assumption that random error is unlikely to occur independently twice at the same restriction sites. We used the Promega enzyme system for two reasons. One was that it allowed for the A- overhang needed for cloning, and also it was the common system in use at the time of data collection (prior to the common availability of the Roche enzyme system). We also included PCR+1 methods into our protocol to insure sequencing of only homoduplex clones, which eliminates issue of chimeric sequences due to heteroduplex formation in PCR. Even the clones produced primarily using the Roche enzyme system had at least one cycle using Promega (PCR+1 cycle, which also added the overhang). While there is some degree of randomness to the polymorphisms observed among sequences, the high degree of conservation of sequence in regions known to be critical to proper function (and are quite highly conserved across species) doesn't support the proposition of high PCR error, which one would expect to be random.

The study has available the data to test the "real genetic variation" vs. "rapid generation of variants" hypotheses. Infection K1 was passed to 6 birds, and KV115 to 5 birds. Thus, we would expect to find the same alleles in all the recipient birds as in the donor bird. Are the same alleles found? Over the course of the infection, are the same alleles found? Or, are different alleles found in the recipients, and do new alleles show up during the infection? There are no such bird-by-bird results presented in the manuscript.

**Authors' response**: We wish we had the samples from the original infections with which to compare. Unfortunately, we don't. We are in the process of trying to complete this type of analysis at this time with more recently derived samples. Thank you for your suggestions.

Researchers working with human malaria parasites would read this paper with great interest, and would conclude that this is a very nice example of the rapid mutation of a surface protein gene, and another example of how these proteins interact with the immune system and are selected to generate variation (analogous to the VAT's in *Trypanosoma*).

Review 2: (Daniel Jeffares Wellcome Trust Sanger Institute, Population and Comparative Genomics Team, Wellcome Trust Genome Campus, Hinxton, Cambridge, CB10 1SA, UK; nominated by Anthony Poole, Arrhenius Laboratories for Natural Sciences, Department of Molecular Biology & Functional Genomics, Stockholm University, SE-106 91 Stockholm Sweden)

In this work Dr. Susan Jarvi and colleagues examine the genetic diversity of the TRAP gene in Hawaiian isolates of the avian malaria parasite *Plasmodium relictum*. They PCR amplify the TRAP gene (which is known to be highly polymorphic in other *Plasmodium *species) from DNA extracts from the blood of 18 birds of various Hawaiian species, clone the products to allow detection of multiple parasite isolates within each bird, and examine the clones by restriction fragment length polymorphism and sequencing.

The study examined TRAP diversity from 7 birds that had been naturally infected with *P. relictum*, 5 birds that had been experimentally infected with one *P. relictum *isolate that had been maintained in laboratory contained mosquitoes, and 6 birds that had been experimentally infected with another laboratory maintained isolate.

The study found multiple TRAP haplotypes in most birds. Since *Plasmodium *parasites are haploid in blood stages this indicates that there is significant genetic diversity in Hawaiian *P. relictum *and that most infections contain multiple parasite clones.

### Main comments

Jarvi et al. clearly show there is significant genetic diversity in Hawaiian *P. relictum *and that most infections contain multiple parasite clones. However, greater understanding of the TRAP gene, or of the population genetics of Hawaiian *P. relictum *will require more data. However the reliable data produced are rather limited, and some of the data analysis is handled inappropriately, in my view. I detail these problems below.

First, the authors own controls indicated that their methods (PCR amplification with a non proof-reading Taq for 80 PCR cycles) produced some PCR-induced polymorphism artefacts. Although PCR-induced polymorphism is not expected to produce patterns such as geographic or phenotypic associations (rather just to add noise), it will increase measures of genetic diversity. The majority of data was produced with the non proof-reading Taq using 80 PCR cycles, however there is no consideration of this in results/discussion sections.

**Authors' response**: We completed this small trial to evaluate these variables in our system. We don't believe that PCR error is significantly impacting estimates of genetic diversity in this study as we have included only clones found independently in two or more separate birds. Please also see previous response to similar concerns of Reviewer 1.

Also, RFLP haplotype data from two birds were effectively removed from the study because they contained unique haplotypes. This will also have the effect of increasing estimates of diversity.

Finally there was a non-random selection of which clones to sequence. Proportionally more clones were chosen from the rarer RFLP haplotypes. This choice will also have the effect of increasing measures of genetic diversity from the sequence data. The result is that while estimates of genetic diversity are high from sequence (genetic diversity =~ 0.06) it is difficult to know how much of this value is artefact, and hence how to compare these values directly to other studies.

**Authors' response**: We removed the birds from the estimates of genetic diversity because of the minimal data collected (only 2 clones and 1 clone were obtained from each of these birds). We did not try to produce more clones from these birds, but kept them in the overall study as we had obtained sequence data from one of them. To include them in the estimates would bias them in the other direction. We sequenced 18 clones from the most common RFLP haplotype (77) and 15 clones representing 12 other haplotypes. I don't believe we disproportionately selected the more rare haplotypes. Since this is not a population-level study, these results may not be directly comparable to other studies.

Another limitation of this study is that apart from the RFLP analysis, the sequence data produced in this study is relatively small. Only 14 TRAP sequences were produced from one of the cultured isolates (10 of which from a single bird infection), 16 from another (again, 10 of which from a single bird infection) and 5 from wild isolates (none of which are derived from high fidelity Taq). The result is that few of the more interesting conjectures in the discussion have significant support. For example there was no significant difference between diversity estimates of the two mosquito-derived isolates, or the wild isolates, nor between those birds that survived experimental infections vs. those that did not. Collection of more sequences from more isolates would allow analysis of the frequency distributions of polymorphisms, and be much more informative. To be fair the authors state that population level genotyping is underway. I would caution readers from drawing too many conclusions until this analysis is available.

For example, I would regard any clustering of TRAP isolates with respect to mortality with strong suspicion. While the TRAP gene *may *contribute to mortality, an association between mortality was not shown (recall that most sequence data are from two isolates from experimentally-infected birds from cultured parasites), and even if it were shown we would need evidence that this association was not due to population subdivision or to linkage with another causal variant.

In summary I think that the data has been extended beyond what it is capable of describing, and may also be limited by PCR-artefacts. Certainly there is no subterfuge about this in the manuscript (the authors for the most part describe the limitations), but the results could well have been limited to a simpler story. Further work on this system, particularly with the improvements that have been shown to be necessary with the data in this work, should provide a much more informative picture.

**Authors' response**: I think it is important to realize that this manuscript is only intended as an initial attempt to begin to genetically characterize diversity in a previously uncharacterized species of *Plasmodium*. It not intended as an analysis of survivors and non-survivors of avian malaria. However, as noted above, many of the individuals under study were involved in experimental infection studies and we feel compelled to include this data here. We have reworked the discussion to reflect this concern. We also included a more detailed description of the isolates. Thank you for your suggestions.

Review 3: (Susan Perkins, American Museum of Natural History, Assistant Curator, Division of Invertebrate Zoology, Central Park West at 79^th ^Street, New York, NY 10024: nominated by Eugene Koonin, Senior Investigator NCBI, NLM, NIH Bethesda, MD 20894)

I found this manuscript, by Jarvi, Farias, and Atkinson to be very interesting and thorough. It also represents an important addition to our knowledge of the nonmammalian and/or non-model system malaria parasites with respect to the genetic diversity that is naturally present in these systems. When I first began reading this manuscript, I immediately worried that some of the high amount of genetic variability might have been caused by heteroduplex formation and bacterial repair during the cloning process, so was very relieved when the authors said that they also incorporated PCR+1 methodology into this study. That said, though, I found it slightly unusual that instead of bumping up the reaction to 80 cycles, they did not also choose to try a scenario of using a very small number of cycles and then pooling these products – a method that is commonly used among microbiologists who are trying to assess diversity in an environment in an attempt to recover rarer organisms (sequences) that might be swamped out by more common ones through the PCR amplification process. My second suggestion is that I think that their sequence data would be far better represented by a haplotype network rather than a traditional, bifurcating tree. As the authors admit, the bootstrap support values on most nodes are very low and this is no doubt a result of a more network-like scenario of evolution that resulted from one or a few haplotypes founding these populations and then accumulating point mutations as the populations expanded and dispersed. A bifurcating, distance-based tree such as the one in Figure 2, will be complicated and distorted by this pattern of evolutionary change. Representing the data as such, along with perhaps shading to depict those sequences found in survivors vs. non-survivors might reveal more interesting and clear-cut patterns than this NJ tree could ever do.

**Authors' response**: Yes, I wish I had thought of that at the time (i.e., pooling reactions using smaller numbers of cycles). Thank you for your suggestion of a haplotype network. We completed this analysis and it is much more informative than a NJ tree. We have incorporated these data into the manuscript.

At times, this paper also seemed like a mixture of what would be two or perhaps even three different publications. There was the central issue of how much genetic diversity of the TRAP gene – and ergo, number of parasite clones, exists within infected birds and whether or not this diversity is related to host or geography. There was also the attempt to look at the correlation of the presence of certain TRAP sequences to survival in infected birds and this was discussed, in part, with particular changes within the TRAP molecule. Finally, there was the embedded study of enzyme, cycle number, and methodology (PCR+1 or not) and their relationships to the number of clones recovered. Perhaps the authors should consider if these three things might not be better split as such to show that first, the optimum method for detecting clonal diversity was found and used and then that the resulting data on the diversity of genotypes was then analyzed in a context to allow for the most powerful ability to correlate virulence with genotype (ideally by sequencing all genotypes present in these infections).

**Authors' response**: Yes, we are packing a lot in a single paper, but individually, the pieces do not seem substantial enough to warrant a publication on their own.

You may want to explain the origin of the K1 and KV115 isolates – these are not "cloned isolates" in the sense that human and rodent malaria researchers are used to thinking about them.

I would advocate for including the primer sequences here, even if they are published elsewhere. This will facilitate the ability of others to use them, if desired, without having to track down multiple other publications, which they may or may not have easy access to.

**Authors' response**: We have included a section describing the origin of the isolates, as well as the sequences of all primers with their references. Thank you for your suggestions.

## Supplementary Material

Additional file 1 Alignment file of predicted amino acid sequence (543 sites) of the 33 sequences produced in this study. Data provided represent the amino acid alignments of the 33 sequences in this study. Included for comparison are *P. relictum *TRAP (AF072818) and *P. gallinaceum *TRAP (U64899). Regions of note are as indicated (repeat assignments apply for *P. relictum *only). Bracketed abbreviations are as follows: {H} Hawaii, {K} Kauai, {S} Survivor, {NS} Non-survivor. Identical sites are indicated as '.', missing sites as '?' and indel sites as '-'. Click here for file
