# Supplementary material for: Genetic characterization of Hawaiian isolates of Plasmodium relictum reveals mixed-genotype infections
Source: Biol Direct. 2008 Jun 25;3:25. doi: 10.1186/1745-6150-3-25 (PMC2464593; doi:10.1186/1745-6150-3-25)
Supplement: Additional file 1 — Alignment file of predicted amino acid sequence (543 sites) of the 33 sequences produced in this study. Data provided represent the amino acid alignments of the 33 sequences in this study. Included for comparison are P. relictum TRAP (AF072818) and P. gallinaceum TRAP (U64899). Regions of note are as indicated (repeat assignments apply for P. relictum only). Bracketed abbreviations are as follows: {H} Hawaii, {K} Kauai, {S} Survivor, {NS} Non-survivor. Identical sites are indicated as '.', missing sites as '?' and indel sites as '-'. [file 1745-6150-3-25-S1.doc]

A Domain

#TP593R.126-{KF} SGSIGYDNWI SYAVPLVYDI VKNLNVSNDG IHLYLSVFTH YLREYIKLGS SLSTNREFAL NIIENLKNKY YLHGSTNLTI

#TP593R.165-{KF} .......... .......... .......... .......... .......... .......... .......... ..........

#TP593R.140-{KF} .......... .......... .......... .......... .......... .......... .......... ..........

#TP593R.134-{KF} .......... .......... .......... .......... .......... .......... .......... ..........

#TP593R.106-{KF} .......... .......... .......... .......... .......... .......... .......... ..........

#TP593R.127-{KF} .......... .......... .......... .......... .......... .......... .......... ..........

#TP593R.101-{KF} .......... .......... .......... .......... .......... .......... .......... ..........

#TP593R.182-{KF} .......... .......... .......... .......... .......... .......... .......... ..........

#TP593R.186-{KF} .......... .......... .......... .......... .......... .......... .......... ..........

#TP623R.175-{HIF} .......... .......... .......... .......... .......... .......... .......... ..........

#TP623R.172-{HIF} .......... .......... .......... .......... .......... .......... .......... ..........

#TP623R.194-{HIF} .......... .......... .......... .......... .......... .......... .......... ..........

#TP623R.186-{HIF} .......... .......... .......... .......... .......... .......... .......... ..........

#TP623R.184-{HIF} .......... .......... .......... .......... .......... .......... .......... ..........

#TP623R.162-{HIF} .......... .......... .......... .......... .......... .......... .......... ..........

#TP623R.114-{HIF} .......... .......... .......... .......... .......... .......... .......... ..........

#TP584.103-{KF} .......... .......... .......... .......... .......... .......... .......... ..........

#TP3186.1-{KW} .......... .......... .......... .......... .......... .......... .......... ..........

#TP1765.8-{HIW} .......... .......... ........G. T......... .......... .......... .......... ..........

#TP2881.2-{KW} .......... .......... .......... .......... .......... .......... .......... ..........

#TP6775.2-{HIW} .......... .......... .......... .......... .......... ....D..... .......... ..........

#TP601.109-{HIF} .......... .......... .......... .......... .......... .......... .......... ..........

#TP620.106-{HIF} .......... .......... .......... .......... .......... .......... .......... ..........

#TP623R.149-{HIF} .......... .......... .....A.... .......... .......... .......... .......... ..........

#TP623R.165-{HIF} .......... .......... .......... .......... .......... .......... .......... ..........

#TP623R.164-{HIF} .......... .......... .......... .......... .......... .......... .......... ..........

#TP736.127-{HIS} .......... .......... .......... .......... .......... .......... .......... ..........

#TP736.103-{HIS} .......... .......... .......... .......... .......... .......... .......... ..........

#TP736.123-{HIS} .......... .......... .......... .......... .......... .......... .......... ..........

#TP736.102-{HIS} .......... .......... .......... .......... .......... .......... .......... ..........

#TP591.101-{KF} .......... .......... .......... .......... .......... ....I..... ...K.S.... ..........

#TP593R.139-{KF} .......... .......... .......... V......... .......... .......... .......... .........V

#TP1145.132-{KS} .......... .......... .......... T..D...... .......... .......... .......... ..........

#AF072818PREL .......... .......... .......... .......... .......... .......... .......... ..........

#PGU64899PGAL ......Y..V T......EE. .Q...I.KQ. .......... I.K...P.N. IF....D... .V.RS.RT.. SQN......L

#TP593R.126-{KF} ALSRVLQDNF I-KKKGREDA VQLILIFTDG APDDKETAMQ EVVKLKKMNA KFSVIGVGMG INREFNKRLV DCSPYEEKCD

#TP593R.165-{KF} .......... .-........ .......... .......... .......... .......... .......... ..........

#TP593R.140-{KF} .......... .-....G... .......... .......... .......... .......... .......... ..........

#TP593R.134-{KF} .......... .-........ .......... .......... .......... .......... .......... ..........

#TP593R.106-{KF} .......... .-........ .......... .......... .......... .......... .......... ..........

#TP593R.127-{KF} .......... .-........ .......... .......... .......... .......... .......... ..........

#TP593R.101-{KF} .......... .-........ .......... .......... .......... .......... .......... ..........

#TP593R.182-{KF} .......... .-........ .......... .......... .......... .......... .......... ..........

#TP593R.186-{KF} .......... .-........ .......... .......... .......... .......... .......... ..........

#TP623R.175-{HIF} .......... .-........ .......... .......... .......... .......... .......... ..........

#TP623R.172-{HIF} .......... .-........ .......... .......... .......... .......... .......... ..........

#TP623R.194-{HIF} .......... .-........ .......... .......... .......... .......... .......... ..........

#TP623R.186-{HIF} .......... .-........ .......... .......... .......... .......... .......... ..........

#TP623R.184-{HIF} .......... .-........ .......... .......... .......... .......... .......... ..........

#TP623R.162-{HIF} .......... .-........ .......... .......... .......... .......... .......... ..........

#TP623R.114-{HIF} .......... .-........ .......... .......... .......... .......... .......... ..........

#TP584.103-{KF} .......... .-........ .......... .......... V......... .......... .......... ..........

#TP3186.1-{KW} .......... .-........ .......... .......... .......... .......... .......... ..........

#TP1765.8-{HIW} .......... .-.Q...... .....T.... .......... .......... .......... .......... ..........

#TP2881.2-{KW} .......... .-........ .......... .......... .........T R......... .......... ..........

#TP6775.2-{HIW} .......... .-........ .......... .......... .......... .......... .......... ..........

#TP601.109-{HIF} .......... .-........ .......... .......... .......... .......... .......... ..........

#TP620.106-{HIF} .......... .-........ .......... .......... .......... .L........ .......... ..........

#TP623R.149-{HIF} .......... .-........ .......... .......... .......... .S........ .......... ..........

#TP623R.165-{HIF} .......... .-........ .......... .......... ....-..... .......... .......... ..........

#TP623R.164-{HIF} .......... .-........ .......... .......... ....-..... .......... .......... ..........

#TP736.127-{HIS} .......... .-........ .......... .......... ....-..... -......... .......... ..........

#TP736.103-{HIS} V......... .-........ .......... .......... .......... .......... .......... ..........

#TP736.123-{HIS} .......... .-........ .......... .......... .......... .......... ....L..... ..........

#TP736.102-{HIS} .......... .-........ .......... ......I... .......... .......... .......... ..........

#TP591.101-{KF} ......L... .-........ .......... .......... .......... .......... .......... ..........

#TP593R.139-{KF} .......... .-........ .......... .......... G......... .......... .......... ..........

#TP1145.132-{KS} .......... .-........ ......I... .......... .......... .......... .......... ..........

#AF072818PREL .......... .-........ .......... .......... .......... .......... .......... ..........

#PGU64899PGAL ......KNY. L-T.GS.... ...VI..... S..N..S..K ..N.....K. ..A....... ..K....S.. G.PLK.K...

Region II

#TP593R.126-{KF} LYSEASWVDV KDIIAPFLKK VCVEIEKVAH CGSWGEWTPC SVTCGEGIKT RKRNILHKGC SDHMNALCEK PECPAIIKPS

#TP593R.165-{KF} .......... .......... .......... .......... .......... .......... .......... ..........

#TP593R.140-{KF} .......... ........E. .......... .......... .......... .......... .......... ..........

#TP593R.134-{KF} .......... .......... .......... .......... .......... .......... .......... ..........

#TP593R.106-{KF} .......... .......... .......... .......... .......... .......... .......... ..........

#TP593R.127-{KF} .......... .......... .........R .......... .......... .......... .......... ..........

#TP593R.101-{KF} .......... .......... ......E... .......... .......... .......... .......... ..........

#TP593R.182-{KF} .......... .......... .......... .......... .......... .......... .......... ..........

#TP593R.186-{KF} .......... .......... .......... .......... .......... .......... .......... ..........

#TP623R.175-{HIF} .......... .......... .......... .......... .......... .......... .......... ..........

#TP623R.172-{HIF} .......... .......... .......... .......... .......... .......... .......... ..........

#TP623R.194-{HIF} .......... .......... .........R .......... .......... .......... .......... ..........

#TP623R.186-{HIF} .......... .......... .......... .......... .......... .......... .......... ..........

#TP623R.184-{HIF} .......... .......... .......... .......... .......... .......... .......... ..........

#TP623R.162-{HIF} .......... .......... .......... .......... .......... .......... .......... ..........

#TP623R.114-{HIF} .......... .......... .......... .......... .......... .......... .......... ..........

#TP584.103-{KF} .......... .......... .......... .......... .......... .R........ .......... ..R.......

#TP3186.1-{KW} .........- .......... ..A....... .......... .......... .......... .......... ..........

#TP1765.8-{HIW} ......R... .......... .......... .......... .......... .......... ........V. ..........

#TP2881.2-{KW} .......... ..V....... .......... ..P....... .......... .......... .......... ..........

#TP6775.2-{HIW} .......... .......... .......... .......... .......... .......... .......... ....?.....

#TP601.109-{HIF} .......... .........E .......... .......... .......... .......... .......... ..........

#TP620.106-{HIF} .......... .........E .......... .E........ .....K.... .......... .......R.. ..........

#TP623R.149-{HIF} .......... .......... .......... .......... .......... .......... .......... ..........

#TP623R.165-{HIF} .......... .......... .......... .......... .......... .......... .......... ..........

#TP623R.164-{HIF} .......... .......... .......... .......... .......... .......... .......... ..........

#TP736.127-{HIS} .......... .......... .......... .......... .......... .......... P......... ..........

#TP736.103-{HIS} .......... .......... .......... .......... .......... .......... .......... ..........

#TP736.123-{HIS} ......?... .......... .......... .......... .......... .......... ........G. ..........

#TP736.102-{HIS} .......... .......... .......... .......... .......... .......... .......... ..........

#TP591.101-{KF} .......... .......... .......... .......... .......... .......... .......... ..........

#TP593R.139-{KF} .......... .......... .......... .......... .......... .......... .......... ..........

#TP1145.132-{KS} .......... .......... .......... .......... .......... .......... ........G. ..........

#AF072818PREL .......... .......... .......... .......... .......... .......... .......... ..........

#PGU64899PGAL .......NE. QNV......E ..I.V..... .......S.. .......VR. .R.EV..... T...TV.... .N..E.V..N

___Repeat1____ _Repeat2_____ __Repeat3___ __Inser_ __Repeat4_____ ___REPEAT5__

#TP593R.126-{KF} VTDIPKVIPE DNRRGDVPDN VPENKKRGDV PDYFPEDNKP LVPDNVPNND PDNAPENKKR GDVPDY-FPE NNQPEVPDNA

#TP593R.165-{KF} .......... .......... .......... .......... .......... .......... ......-... ..........

#TP593R.140-{KF} .......... .......... .......... .......... .......... .......... ......-... ..........

#TP593R.134-{KF} .......... .......... .......... .......... .......... .......... ......-... ..........

#TP593R.106-{KF} .......... .......... .......... .......... .......... .......... ......-... ..........

#TP593R.127-{KF} .......... .......... .......... .......... .......... .......... ......-... ..........

#TP593R.101-{KF} .......... .......... .......... .......... .......... .......... ......-... ..........

#TP593R.182-{KF} .......... .......... .......... .......... .......... .......... ......-... ..........

#TP593R.186-{KF} .......... .......... .......... -......... .......... .......... ..A...-... ..........

#TP623R.175-{HIF} .......... .......... .......... .......... .......... .......... ......-... ..........

#TP623R.172-{HIF} .......... .......... .......... .......... .......... .......... ......-... ..........

#TP623R.194-{HIF} .......... .......... .......... .......... .......... .......... ......-... ..........

#TP623R.186-{HIF} .......... .......... .......... .......... .......... .......... ......-... ..........

#TP623R.184-{HIF} .......... .......... .......... .......... .......... .......... ......-... ..........

#TP623R.162-{HIF} .......... .......... .......... .......... .......... .......... ......-... ..........

#TP623R.114-{HIF} .......... .......... .......... .......... .......... .......... ......-... ..........

#TP584.103-{KF} .......... .......... .......... -......... .......... .......... ......-... ..........

#TP3186.1-{KW} .......... .......... .......... .......... .......... .......... ......-... ..........

#TP1765.8-{HIW} .......... .......... .......... .......... .......... .......... ......-... ..........

#TP2881.2-{KW} .......... .......... .......... ......V... .......... .......... ......-... ..........

#TP6775.2-{HIW} .......... .......... .......... .......... .......... .......E.. ......-... ..........

#TP601.109-{HIF} .......... .......... .....R.... .......... .......... .......... ......-... ..........

#TP620.106-{HIF} .......... .......... .......... .......... .......... .......... ......-... ..........

#TP623R.149-{HIF} .......... .......... .......... .......... .......... .......... ......-... ..........

#TP623R.165-{HIF} .......... .......... .......... .......... ........D. .......... ......-... ..........

#TP623R.164-{HIF} .......... .......... .......... .......... ........D. .......... ......-... ..........

#TP736.127-{HIS} .......... .......... .......... .......... .......... .......... ......-... ..........

#TP736.103-{HIS} .......... .......... .......... .......... .......... .......... ......-... ..........

#TP736.123-{HIS} .......... .......... .......... .......... .......... .......... ......-... ..........

#TP736.102-{HIS} .......... .......... .......... .......... .......... .......... ......-... ..........

#TP591.101-{KF} .......... .......... .......... .......... .......... .......... ......-... ..........

#TP593R.139-{KF} .......... .......... .......... .......... .......... .......... ......-... ..........

#TP1145.132-{KS} .......... .......... .......... .......... .......... .......... ......-... ..........

#AF072818PREL .......... .......... .......... .......... .......... .......... ......-... ..........

#PGU64899PGAL I..V.D.-.D --EPEPI.EE KKPEPVPEEK KPESAPEE.N PESVPEEKKP ESVPE.KEPE SVPEEKEPES VPEEKE.ES.

__Repeat6___ __Repeat7___ ___Repeat8__ ___Repeat9___

#TP593R.126-{KF} PEDNQPEVPD NVPEENQPEV PYNVPEENQP EVPDNVPEEN QPEVPDNVPE DRNPEIPEEK KPENIPENRK EEIIEYIPKN

#TP593R.165-{KF} .......... .......... .......... .......... .......... .......... .......... ..........

#TP593R.140-{KF} .......... .......... .......... .......... .......... .......... .......... ..........

#TP593R.134-{KF} .......... .......... .......... .......... .......... .......... .......... ..V.......

#TP593R.106-{KF} .......... .......... .......... .......... .......... .......... .......... ..........

#TP593R.127-{KF} .......... .......... .......... .......... .......... .......... .......... ..........

#TP593R.101-{KF} .......... .......... .......... .......... .......... .......... .......... ....G.....

#TP593R.182-{KF} .......... .......... .......... .......... .......... .......... .......... ..........

#TP593R.186-{KF} .......... .......... .......... .......... .......... .......... .......... ..........

#TP623R.175-{HIF} .......... .......... .......... .......... .......... .......... .......... ..........

#TP623R.172-{HIF} .......... .......... .......... .......... .......... .......... .......... ..........

#TP623R.194-{HIF} .......... .......... .......... .......... .......... .......... .......... ..........

#TP623R.186-{HIF} .......... .......... .......... .......... .......... .......... .......... ..........

#TP623R.184-{HIF} .......... .......... .......... .......... .......... .......... .......... ..........

#TP623R.162-{HIF} .......... .......... .......... .......... .......... .......... .......... ..........

#TP623R.114-{HIF} .......... .......... .......... .......... .......... .......... .......... ..........

#TP584.103-{KF} .......... .......... .......... .......... .......... V......... .......... ..........

#TP3186.1-{KW} S......... .......... .......... .......... .......... .......... .......... ..........

#TP1765.8-{HIW} .......... .......... .......... .......... .......... .......... .......... ..........

#TP2881.2-{KW} ..G....... ....G..... .......... .......... .......... .......... .......... ..........

#TP6775.2-{HIW} .......... ..T...R.V. .......... .......... .......... .......... .......... ..........

#TP601.109-{HIF} .......... .......... .......... .......... .......... .......... .......... ..........

#TP620.106-{HIF} .......... .......... .......... .......... .......... .....F.... .......... ..........

#TP623R.149-{HIF} .......... .......... .......... .......... .......... .......... .......... ..........

#TP623R.165-{HIF} .......... .......... ........R. .......... .......... .......... .......... ..V.......

#TP623R.164-{HIF} .......... .......... ........R. .......... .......... .......... .......... ..V.......

#TP736.127-{HIS} .......... .......... .......... .......... .......... .......... .......... ..........

#TP736.103-{HIS} .......... .......... .......... .......... .......... .......... .......... ..........

#TP736.123-{HIS} .......... .......... ......G... .......... .........G .......... .......... ..........

#TP736.102-{HIS} .......... .......... .......... .......... .......... .......... .......... ..........

#TP591.101-{KF} ....L..... .......... .......... ....S..... .......... .......... .......... ....V.....

#TP593R.139-{KF} .......... .......... .......... .......... .......... .......... .......... ..........

#TP1145.132-{KS} .......... .......... .......... .......G.. .......... .......... .......... ..........

#AF072818PREL .......... .......... .......... .......... .......... .......... .......... ..........

#PGU64899PGAL ..EKK..SDP EEKKLEPIPE GKKIEPIPEE .KLEPI...K K..SV----. ..ES.PVPDG EA..V.Q.IP DDEQ.EKISG

#TP593R.126-{KF} IPDDVEILPN ENPRIIIKDQ RHLPPQVVPP KNIHNENQII NKVPEHNGNI NKTTVEDREL RPHNTDNEYI RPRRNDYKVE

#TP593R.165-{KF} .......... .......... .......... .......... .......... .......... .......... ..........

#TP593R.140-{KF} .......... .......... .......... .......... .......... .......... .......... ..........

#TP593R.134-{KF} .......... .......... .......... .......... .......... .......... .......... ..........

#TP593R.106-{KF} .......... .......... .R........ .......... .......... .......... .......... ..........

#TP593R.127-{KF} .......... .......... .......... .....K.... .......... .......... .......... ..........

#TP593R.101-{KF} .......... .......... .......... ..V....... ......S... .......... .......... ..........

#TP593R.182-{KF} .......... .......... .......... .......... .......... .......... .......... ..........

#TP593R.186-{KF} .......... .......... .......... .......... .......... .......... .......... ..........

#TP623R.175-{HIF} .......... .......... .........A .......... .......... .......... .......... ..........

#TP623R.172-{HIF} .......... .......... .........A .......... .......... .......... .......... ..........

#TP623R.194-{HIF} .......... .......... .........A .......... .......... .......... ...S...... ..........

#TP623R.186-{HIF} .......... .......... .........A .......... .......... .......... .......... ..........

#TP623R.184-{HIF} .......... .......... .........A .......... .......... .......... .......... ..........

#TP623R.162-{HIF} .......... .......... .........A .......... .......... .......... .......... ..........

#TP623R.114-{HIF} .......... .......... .........A .......... .......... .......... .......... ..........

#TP584.103-{KF} .......... .....T.... .......... .......... .......... .......... .......... ..........

#TP3186.1-{KW} .......... ...G...... .......... .......... .......... .......... .........V ..........

#TP1765.8-{HIW} .......... ..S....... .......... .......... .R........ .......... .......... ..........

#TP2881.2-{KW} .......... .......... ......A..A .......... .......... .......... S.......C. ..........

#TP6775.2-{HIW} .......... .......... .........A .......... .......... .......... .......... ..........

#TP601.109-{HIF} .......... .......... .........A ..T....... .........V .......... .......... ..........

#TP620.106-{HIF} .......... .......... .........A .......... ....G..... .......... .......... ..........

#TP623R.149-{HIF} .......... .......... .........A .......... .......... .......... .......... ..........

#TP623R.165-{HIF} .......... .......... .........A .......... .......... .......... .......... ..........

#TP623R.164-{HIF} .......... .......... .........A .......... .......... .......... .......... ..........

#TP736.127-{HIS} .......... .......... .........A .......... .......... .......... .......... ..........

#TP736.103-{HIS} .......... .S........ .........A .......... .......... .......... .......... ..........

#TP736.123-{HIS} ......L... .......... .........A .......... .......... .......... .......... ..........

#TP736.102-{HIS} .......... .......... .........A .......... .......... .......... .......... .........K

#TP591.101-{KF} .......... .......... .......... .......... .......... .........P .......... ..........

#TP593R.139-{KF} .......... .......... .......... .......... .......... .......... .......... ..........

#TP1145.132-{KS} .......... .......... .......... .......... .........V .......... .......... ..........

#AF072818PREL .......... .......... .........A .......... .......... .......... .......... ..........

#PGU64899PGAL DIPND.E.IP K.EPDD..RN EYDTTPNII. PKDTYNDNE. TNPISEED.E ...K....VP ......S... P.K.DNH.D.

Transmembrane Region

#TP593R.126-{KF} PSTENVENEN SEEKNKKA-- RDNKYKIAGG IIGGLALLGC AGFAYKFLAH APTPPMTSEG APF

#TP593R.165-{KF} .......... ........-- S......... .......... .......... .......... ...

#TP593R.140-{KF} .......... ........-- S......... .......... .......... .......... ...

#TP593R.134-{KF} .......... ........-- S......... .......... .......... .......... ...

#TP593R.106-{KF} .......... ........-- S......... .......... .......... .......... ...

#TP593R.127-{KF} .......... ........-- S......... .......... .......... .......... ...

#TP593R.101-{KF} .......... ....ST..-- S......... .......... .......... .......... ...

#TP593R.182-{KF} .......... ........-- S......... .......... .......... .......... ...

#TP593R.186-{KF} .......... ........-- S......... .......... .......... .......... ...

#TP623R.175-{HIF} .......... ........-- S......... .......... .......... .......... ...

#TP623R.172-{HIF} .......... ........-- S......... .......... .......... .......... ...

#TP623R.194-{HIF} .......... ........-- S......... .......... .......... .......... ...

#TP623R.186-{HIF} .......... ........-- S......... .......... .......... .......... ...

#TP623R.184-{HIF} .......... ..-.....-- S......... .......... .......... .......... ...

#TP623R.162-{HIF} .......... ........-- S......... .......... .......... .......... ...

#TP623R.114-{HIF} .......... ........-- S......... .......... .......... .......... ...

#TP584.103-{KF} .......... ........-- S......... .......... .......... .......... ...

#TP3186.1-{KW} .......... ........-- S......... .......... .......... .......P.. ...

#TP1765.8-{HIW} .......... ........-- S......... .......... .......S.. .......... ...

#TP2881.2-{KW} .......... F.......-- S......... .......... .......... .......... ...

#TP6775.2-{HIW} .......... ........-- S......... .......... .......... .......... ...

#TP601.109-{HIF} .......... ........-- S......... .......... .......... .......... ...

#TP620.106-{HIF} .......D.. F.......-- S......... .......... .......... .......... ...

#TP623R.149-{HIF} .......... ........-- S......... .......... .......... .......... ...

#TP623R.165-{HIF} .......... ........-- S......... .......... .......... .......... ...

#TP623R.164-{HIF} .......... ........-- S......... .......... .......... .......... ...

#TP736.127-{HIS} .......... ........-- S......... .......... .......... .......... ...

#TP736.103-{HIS} .......... ........-- S......... .......... .....E.... .......... ...

#TP736.123-{HIS} .......... ........-- S......... .......... .......... .......P.. ...

#TP736.102-{HIS} .......... P.......-- S......... .......... .......... ......A... ...

#TP591.101-{KF} .......... ........-- S......... .......... .......... .......... ...

#TP593R.139-{KF} .......... ........-- S......... .......... .......... .......... ...

#TP1145.132-{KS} R......... ........-- S......... .......... ......S... .......... ...

#AF072818PREL .......... ........-- S......... .......... .......... .......... ...

#PGU64899PGAL ..RRKR...G TQG.T..TSL N......... .......... ........TQ T....I...A ...
